# Genome-Wide Identification of Terpene Synthase Genes in *Siraitia grosvenorii* Reveals Sexual Dimorphism in Floral Traits and a Fruit-Specific Candidate *SgTPS49*

**DOI:** 10.3390/genes17080926

**Published:** 2026-08-06

**Authors:** Xiaozhen Zhu, Qifeng Lu, Changqiu Liu, Xinghua Hu, Jiatong Ye, Tao Deng, Yunbo Duan, Yufeng Wang

**Affiliations:** Guangxi Key Laboratory for Plant Functional Phytochemicals and Sustainable Utilization, Guangxi Institute of Botany, Guangxi Zhuang Autonomous Region and Chinese Academy of Sciences, Guilin 541006, China; zhuxiaozhen16@163.com (X.Z.); luiqfeng@163.com (Q.L.); changqiuliu@whu.edu.cn (C.L.); cate_yejt@163.com (J.Y.); qbtao166@163.com (T.D.); yunboduan@126.com (Y.D.); wyf03170517@163.com (Y.W.)

**Keywords:** *Siraitia grosvenorii*, terpene synthase, gene family, fruit development, sexual dimorphism, floral volatiles, tandem duplication, synteny analysis

## Abstract

Background: *Siraitia grosvenorii* (monk fruit) is a dioecious medicinal crop native to southern China, yet its terpene synthase (TPS) gene family and the molecular basis of floral sexual dimorphism remain unexplored. Methods: Genome-wide identification of the TPS gene family was performed using HMMER and BLAST-based approaches. Phylogenetic classification, gene structure and conserved motif characterization, and comparative synteny analyses were conducted. Transcriptome data from leaves and fruits at different developmental stages were analyzed for tissue-specific expression profiling. Promoter cis-element analysis, protein–protein interaction network prediction, and molecular docking were performed to characterize the fruit-specific candidate *SgTPS49*. Results: A total of 58 SgTPS genes were identified and classified into six subfamilies, with TPS-a and TPS-b comprising 72.4% of the family. Approximately 88% of SgTPS genes arose from lineage-specific tandem duplication. Female flowers exhibited monoterpene-dominant scents and smaller corollas, whereas male flowers displayed a mid-morning sesquiterpene burst and greater morphological variation. *SgTPS49* was specifically upregulated at 20 days post-pollination and possessed a unique promoter architecture devoid of classical hormone-responsive elements. Molecular docking supported its annotation as a putative monoterpene synthase with favorable GPP binding. Conclusions: This study provides the first comprehensive genomic resource for the SgTPS family in *S. grosvenorii*, reveals significant sexual dimorphism in floral traits, and identifies *SgTPS49* as a key candidate for future functional validation.

## 1. Introduction

Terpene synthases (TPSs) are rate-limiting enzymes that drive the biosynthesis of structurally diverse mono-, sesqui-, and diterpene skeletons from isopentenyl pyrophosphate and dimethylallyl diphosphate precursors [1]. TPS proteins typically contain conserved motifs, such as the RRX_8_W motif for substrate recognition and the DDXXD and NSE/DTE motifs required for catalytic activity and metal ion binding [2,3]. Systematic analyses across plant species have revealed marked differences in TPS family size and evolutionary patterns. For example, 32 TPS genes have been reported in *Arabidopsis thaliana* [2], 44 in tomato [4,5], and variable numbers in other horticultural crops [6]. In tomato, the TPS-a subfamily member *SlTPS5* is highly expressed during fruit ripening, contributing directly to the production of volatile sesquiterpenes and fruit aroma [4]. This principle, where the precise spatial and temporal expression of TPS genes acts as a critical determinant of fruit quality traits such as flavor and aroma, is well-established in model systems [5]. Recent advances in bioinformatics have enabled comprehensive characterization of TPS gene families in diverse plant species [7,8,9,10,11,12,13], including medicinal and aromatic plants such as *Liriodendron chinense* [14], *Chenopodium quinoa* [15], and *Dendrobium catenatum* [16], consistently highlighting lineage-specific expansion as a major evolutionary force driving terpenoid diversity. Notably, terpenoid diversity is shaped not only by gene family expansion but also by the catalytic promiscuity and subcellular compartmentalization of TPS enzymes, as comprehensively reviewed by Jia et al. [17]. In the Cucurbitaceae, however, most research has focused on a limited number of species, and systematic studies remain scarce for medicinal plants such as *Siraitia grosvenorii*, which produces unique mogrosides [18]. Previous studies on cucurbit TPS genes, such as the functional characterization of the cucumber (*Cucumis sativus*) TPS family [19] and the analysis of sesquiterpene biosynthesis in melon (*Cucumis melo*) [20], have provided valuable insights into terpenoid metabolism in this family, yet the TPS repertoire of monk fruit remains completely unexplored.

*S. grosvenorii* (Luohanguo, or monk fruit) is a dioecious species of Cucurbitaceae native to southern China [21]. Its fruit contains mogrosides, highly sweet triterpene saponins of notable medicinal and economic value [18,22]. Although high-quality draft and haplotype-resolved genomes are available [23,24], the SgTPS gene family has not been systematically studied. The CuGenDBv2 database provides essential genomic resources for comparative studies [25], and recent surveys have identified other gene families, such as *ACS* genes involved in gender differentiation [26], reflecting expanding resources for this species. Mogroside biosynthesis relies on farnesyl diphosphate (FPP), which is also used by members of the TPS-a subfamily to produce sesquiterpenes. We hypothesize that high TPS-a expression may divert FPP from mogroside biosynthesis, potentially contributing to unstable mogroside content in cultivation [24].

As a dioecious plant, *S. grosvenorii* exhibits pronounced sexual dimorphism in floral morphology and scent, a phenomenon widely documented in dioecious species [27], both of which are important for pollinator attraction and reproductive success [28,29]. The biosynthesis and emission of floral terpenoids are mediated by spatially and temporally coordinated TPS enzymes, whose expression patterns directly shape the sex-specific volatile profiles observed in dioecious species, as demonstrated by TPS-mediated floral scent evolution in *Aquilegia* [30] and sex-specific volatile variation in *Schiedea globosa* [31]. Transcriptomic analyses have identified genes involved in gender differentiation, clarifying the molecular basis of this dimorphism [26]. However, the connection between sex-specific floral volatiles and TPS gene expression remains largely unstudied. Understanding this link will provide deeper insight into the species’ reproductive biology and secondary metabolism.

This study aimed to: (1) compare morphological and volatile differences between female and male flowers; (2) identify and characterize the SgTPS gene family at the genome-wide level; (3) analyze tissue-specific SgTPS gene expression during fruit development; (4) predict the structure and substrate-binding mode of the fruit-specific *SgTPS49* through homology modeling and molecular docking; (5) assess the relationship between promoter cis-elements and SgTPS gene expression; and (6) investigate the evolutionary origin of the SgTPS family using comparative synteny analysis. These results will inform future functional studies of *SgTPS49* and advance understanding of terpenoid metabolism in *S. grosvenorii*.

## 2. Materials and Methods

### 2.1. Plant Materials and Floral Morphology Measurement

Female and male *S. grosvenorii* plants were cultivated in Yongfu County, Guilin City, China, under standard field management practices. Fully opened flowers from male (*n* = 30) and female (*n* = 15) plants were harvested for morphological measurements using a digital caliper (accuracy 0.01 mm; Guanglu Digital Measuring Co., Ltd., Guilin, China). Data are reported as mean ± standard deviation (SD). For molecular analyses, tissues including roots, stems, leaves, female and male flowers, and fruits at 3 and 20 days post-pollination (DPP) were collected, immediately flash-frozen in liquid nitrogen, and stored at −80 °C. These materials remain available for future validation experiments, including quantitative real-time PCR (qRT-PCR). *S. grosvenorii* is widely cultivated and is not classified as a rare or endangered species. Voucher specimens have been deposited in the Herbarium of Guangxi Institute of Botany (IBK), barcode IBK00116376. Morphological and volatile data used in this study were originally generated for a previous master’s thesis [32]. For this manuscript, all raw data were re-analyzed, new statistical tests were performed, and all figures regenerated. The interpretations and integration with genome-wide TPS analysis are novel to this work.

### 2.2. Floral Volatile Collection and GC–MS Analysis

Floral volatiles were collected at 6:00, 8:00, 10:00, 12:00, 14:00, 16:00, and 18:00 using headspace solid-phase microextraction (HS-SPME) with a 30 μm PDMS/DVB fiber (Supelco, Bellefonte, PA, USA) and analyzed by gas chromatography–mass spectrometry (GC–MS; Agilent 7890C gas chromatograph coupled with an Agilent 5975 mass spectrometer, Agilent Technologies, Santa Clara, CA, USA) [33]. Separation was performed on an HP-5MS capillary column (30 m × 0.25 mm × 0.25 μm; Agilent Technologies, Santa Clara, CA, USA) with the following oven program: 40 °C for 3 min, increased to 250 °C at 5 °C min^−1^, and held for 5 min. Compounds were identified by matching mass spectra with the NIST 05a.L library (National Institute of Standards and Technology, Gaithersburg, MD, USA), and only compounds with a match factor ≥ 80% were retained for quantification. Compound identities are provided with CAS numbers in Supplementary Table S3. Three biological replicates were performed for each time point and sex.

### 2.3. Genome-Wide Identification and Characterization of SgTPS Genes

The genome assembly, protein sequences, and GFF3 annotation files of *S. grosvenorii* were obtained from the improved genome published by Xia et al. [23]. Raw sequencing reads are available from the Genome Sequence Archive (accession CRA000522, https://ngdc.cncb.ac.cn/gsa, accessed on 5 January 2026) and from the European Nucleotide Archive (BioProjects PRJEB23465 and PRJEB23466, https://www.ebi.ac.uk/ena, accessed on 5 January 2026). The assembly is approximately 469.5 Mb with a contig N50 of 432 kb and contains 30,565 predicted protein-coding genes [23]. The cucurbit genomics database, CuGenDBv2, was also consulted for comparative analyses [25]. To identify TPS family members, two complementary approaches were employed. First, TPS protein sequences from *A. thaliana* and rice were used as queries for TBLASTN and BLASTP (https://blast.ncbi.nlm.nih.gov, accessed on 5 January 2026) against the *S. grosvenorii* genome and proteome with an E-value threshold of 1 × 10^−5^ [3]. Second, HMMER v3.3.2 was used to search the proteome with the Pfam hidden Markov models for the TPS N-terminal domain (PF01397) and C-terminal domain (PF03936), also with an E-value threshold of 1 × 10^−5^. Candidate proteins containing both domains were retained and validated using the NCBI CDD tool (https://www.ncbi.nlm.nih.gov/cdd, accessed on 5 January 2026). The identified SgTPS genes were designated *SgTPS1* to *SgTPS58* according to their chromosomal locations. Physicochemical properties were computed using ProtParam (https://web.expasy.org/protparam/, accessed on 5 January 2026), and subcellular localization was predicted using WoLF PSORT (https://wolfpsort.hgc.jp/, accessed on 5 January 2026).

### 2.4. Phylogenetic Analysis, Gene Structure, and Conserved Motifs

Multiple sequence alignment of the 58 SgTPS proteins was performed using ClustalW in MEGA 11 (v11.0.13; https://www.megasoftware.net/, accessed on 8 January 2026). A maximum-likelihood (ML) phylogenetic tree was constructed using the best-fit model, LG + G + I, with 1000 bootstrap replicates, and visualized with iTOL (https://itol.embl.de/, accessed on 8 January 2026). For multi-species phylogenetic analysis, TPS protein sequences from *A. thaliana*, rice, tomato, cucumber, and *S. grosvenorii* were aligned, and a neighbor-joining tree was constructed using the Poisson model with 1000 bootstrap replicates in MEGA 11. The resulting tree was visualized as a circular phylogram using iTOL to illustrate the evolutionary relationships among TPS genes from representative monocot (rice), dicot (*Arabidopsis* and tomato), and Cucurbitaceae (cucumber and *S. grosvenorii*) species. Exon–intron structures were extracted from the GFF3 file and visualized using TBtools-II (v2.441) [34]. Conserved motifs were identified using MEME Suite (https://meme-suite.org/, accessed on 8 January 2026) with the parameters: maximum number of motifs = 10, minimum width = 6, maximum width = 50.

### 2.5. Promoter Cis-Regulatory Element Analysis

The 2000 bp sequences upstream of the start codon of each SgTPS gene were extracted as promoter regions using TBtools-II (v2.441) [34] and submitted to PlantCARE for cis-regulatory element prediction [35]. This region was selected because it captures most functionally validated cis-regulatory elements in plant gene promoters while limiting the inclusion of distal non-regulatory sequences. Results were categorized by function, including hormone responsiveness, stress responsiveness, light responsiveness, and development-related elements, and visualized using TBtools-II (v2.441) [34].

### 2.6. Expression Analysis of SgTPS Genes

Transcriptome data for leaves, fruits at 3 DPP, and fruits at 20 DPP were obtained from Xia et al. [23] under ENA BioProject PRJEB23466 (https://www.ebi.ac.uk/ena, accessed on 12 January 2026). Raw reads were quality-filtered using fastp v0.24.1, mapped to the *S. grosvenorii* genome using HISAT2 v2.2.1, and gene expression levels (FPKM) were calculated using StringTie v2.2.1. Genes with FPKM values below 1 across all samples were filtered out, leaving 33 SgTPS genes. FPKM values were Z-score normalized and visualized as a heatmap using TBtools-II (v2.441) [34]. To identify stage-preferential genes, an empirical fold-change criterion was applied: genes were considered differentially expressed between the two conditions if the mean FPKM across the two biological replicates changed by more than 2-fold (|log_2_FC| > 1), and the mean FPKM in at least one condition was ≥ 1. For integrated promoter–expression analysis, Z-score normalization was applied to the mean FPKM values of the 33 detectably expressed genes across the three tissues, and a heatmap was generated using TBtools-II (v2.441) [34].

### 2.7. Protein–Protein Interaction Network Prediction for SgTPS49

To predict the functional protein association network of SgTPS49, its protein sequence was used as a query in BLASTP against the *A. thaliana* proteome with an E-value threshold of 1 × 10^−10^. The top homologous gene was AT1G23870.1. The TAIR gene ID of this homolog was submitted to the STRING database v11.5 (https://string-db.org/, accessed on 15 January 2026). The interaction network was retrieved with a minimum required interaction confidence score of 0.70. Following the method described in Szklarczyk et al. [36], the network was visualized using Cytoscape v3.9.1.

### 2.8. Tandem Duplication Analysis

Chromosomal locations of all 58 SgTPS genes were extracted from the GFF3 annotation. Genes were considered tandemly duplicated if they were located on the same contig, adjacent to each other with no other SgTPS gene in between, and separated by less than 50 kb [37]. The distances between adjacent gene start positions were calculated manually from the sorted list of coordinates. The resulting tandem arrays were recorded and classified by subfamily membership.

### 2.9. Statistical Analysis

Morphological measurements were analyzed using Student’s *t*-test in R 4.5.3 (R Core Team, 2024) with the ggpubr package for visualization. For volatile data, three biological replicates were used; significant differences between female and male at each time point were assessed by Student’s *t*-test followed by false discovery rate (FDR) correction using the Benjamini–Hochberg procedure. An FDR-adjusted *p*-value < 0.05 was considered statistically significant. Data are presented as mean ± SD.

### 2.10. Homology Modeling and Molecular Docking

The three-dimensional structure of SgTPS49 was predicted using SWISS-MODEL [38] (https://swissmodel.expasy.org/, accessed on 18 January 2026) based on its full-length amino acid sequence. The template with the highest sequence identity, the AlphaFold DB model of *Cucurbita moschata* nerolidol synthase 1-like (A0A6J1EK53.1.A) with 72.47% identity and a GMQE of 0.50, was selected. Molecular docking of the substrate geranyl diphosphate (GPP) was performed using the CB-Dock2 server [39] (https://cadd.labshare.cn/cb-dock2/, accessed on 18 January 2026), which employs AutoDock Vina 1.2.7 for cavity-guided blind docking. The binding cavity with the lowest Vina score was considered the most probable active site. Visualization of the protein–ligand complex was performed with Mol* [40] and PyMOL 3.1.0.

### 2.11. Synteny Analysis

To investigate the evolutionary conservation and origin of the SgTPS gene family, genome-wide synteny analyses were performed between *S. grosvenorii* and two representative cucurbit species, *C. sativus* (cucumber, Chinese Long v3) and *Benincasa. hispida* (wax gourd, B227 v1). Genome assemblies, protein sequences, and GFF3 annotation files for all three species were obtained from the Cucurbit Genomics Database (CuGenDBv2, http://www.cucurbitgenomics.org/, accessed on 22 January 2026). Syntenic blocks were identified using MCScanX (V1.0.0) [41] with an E-value threshold of 1 × 10^−5^ and a maximum gap of 25 genes. The SgTPS gene IDs were supplied as a highlight list, and synteny maps were visualized using TBtools-II (v2.441) [34].

## 3. Results

### 3.1. Sexual Dimorphism in Floral Volatile Emissions

GC–MS analysis showed marked differences in volatile profiles between female and male flowers. Female flowers emitted a relatively simple scent profile dominated by monoterpenes, particularly α-pinene and β-pinene, which together accounted for the majority of the total volatile content across all time points. β-Pinene reached its highest relative content of 50.18% at 6:00, whereas α-pinene peaked later at 10:00 at 51.86% (Supplementary Figure S1), indicating a temporal shift in the dominant monoterpene over the course of the morning. In contrast, male flowers released a significantly more diverse and complex volatile mixture. In addition to monoterpenes such as α-pinene, myrcene, and dipentene, male flowers emitted substantially higher levels of sesquiterpenes, including α-cubebene and α-copaene, with the latter reaching 67.60% of total volatiles at 8:00. At most time points, the relative contents of major compounds differed significantly between sexes (FDR-adjusted *p* < 0.05; Supplementary Table S3). Notably, linalool, a monoterpene alcohol commonly associated with floral scent in many plant species, was not detected in any of the headspace volatile samples from either female or male flowers across the seven time points (Supplementary Table S3).

**Figure 1:**
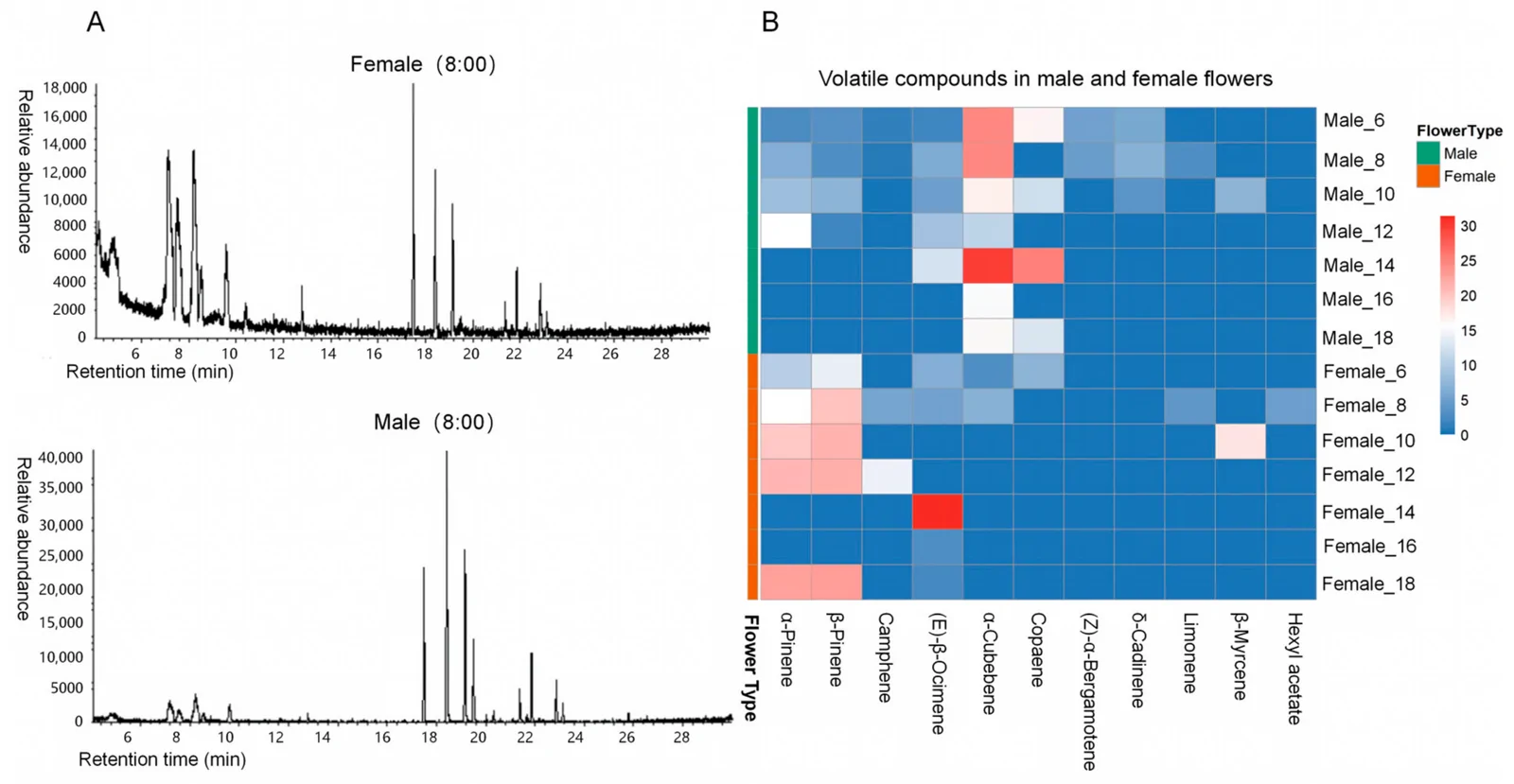
Volatile profiles of female and male *Siraitia grosvenorii* flowers. (**A**) Representative total ion chromatograms at 8:00 from female (top) and male (bottom) flowers. (**B**) Dynamic changes in total monoterpene and sesquiterpene relative contents (6:00–18:00). Red lines, female; blue lines, male; solid lines, monoterpenes; dashed lines, sesquiterpenes. Data are mean ± SD of three biological replicates.

Temporal profiling of total monoterpene and sesquiterpene contents at seven time points revealed clear differences in scent-emission patterns between the sexes (Figure 1B). Female flowers maintained consistently high monoterpene levels throughout the day, while sesquiterpene content remained low and stable, resulting in a chemically simple and predictable scent profile. In contrast, male flowers showed a distinct burst of sesquiterpene emission in the mid-morning, coinciding with peak insect activity. This sesquiterpene pulse, layered over moderate monoterpene levels, produced a dynamic and complex scent profile. The greater variation in male volatile composition, reflected by larger standard deviations in Figure 1B, indicates a higher degree of phenotypic plasticity in male floral scent. Comparable patterns of sex-biased volatile production have been reported in other dioecious species and are often linked to differences in pollinator attraction strategies [28,29,31].

### 3.2. Sexual Dimorphism in Floral Morphology

**Figure 2:**
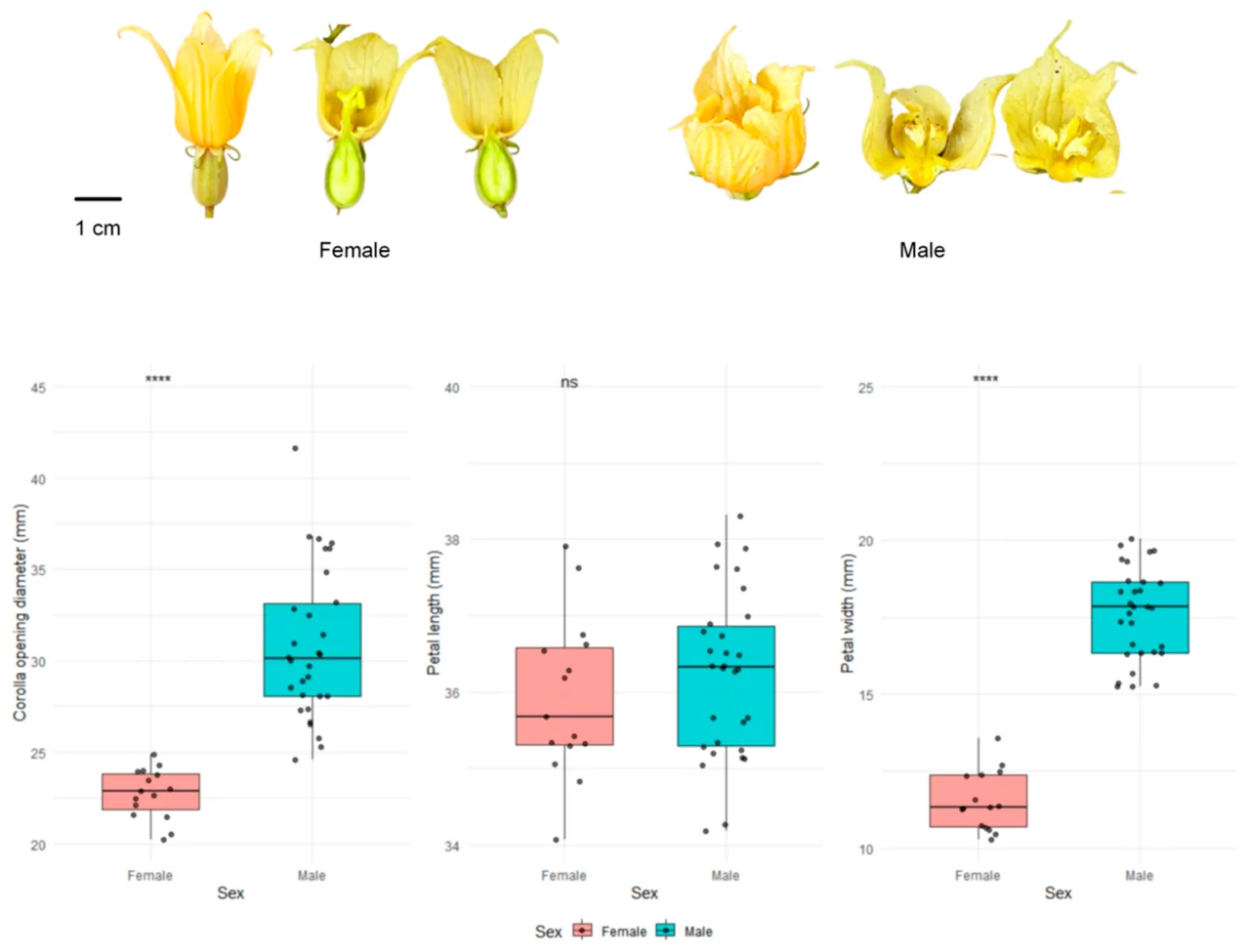
Morphological comparison of female and male *S. grosvenorii* flowers. (**Top**) Intact flower and dissected floral organs of female (left three panels) and male (right three panels). (**Bottom**) Boxplots of corolla opening diameter, petal length and petal width. **** *p* < 0.0001; ns, not significant. Female: *n* = 15. Male: *n* = 30.

Female and male flowers of *S. grosvenorii* showed pronounced morphological differences across multiple traits. The corolla opening diameter, a trait directly associated with pollinator access, was significantly smaller in female flowers than in males (22.90 ± 1.34 mm vs. 30.82 ± 4.06 mm, *p* < 0.0001; *n* = 15 and 30, respectively; Table 1). Notably, males exhibited considerably greater variation in corolla opening, with a standard deviation nearly threefold that of females (4.06 vs. 1.34 mm), indicating greater morphological variation in male flowers. Petal width also differed markedly between sexes (11.57 ± 0.98 mm in females vs. 17.60 ± 1.47 mm in males, *p* < 0.0001), additionally adding to the larger overall floral display of male flowers (Figure 2). In contrast, petal length was remarkably conserved between sexes (35.96 ± 1.14 mm vs. 36.27 ± 1.27 mm, *p* = 0.43), suggesting that this trait is relatively conserved between sexes.

**Figure 3:**
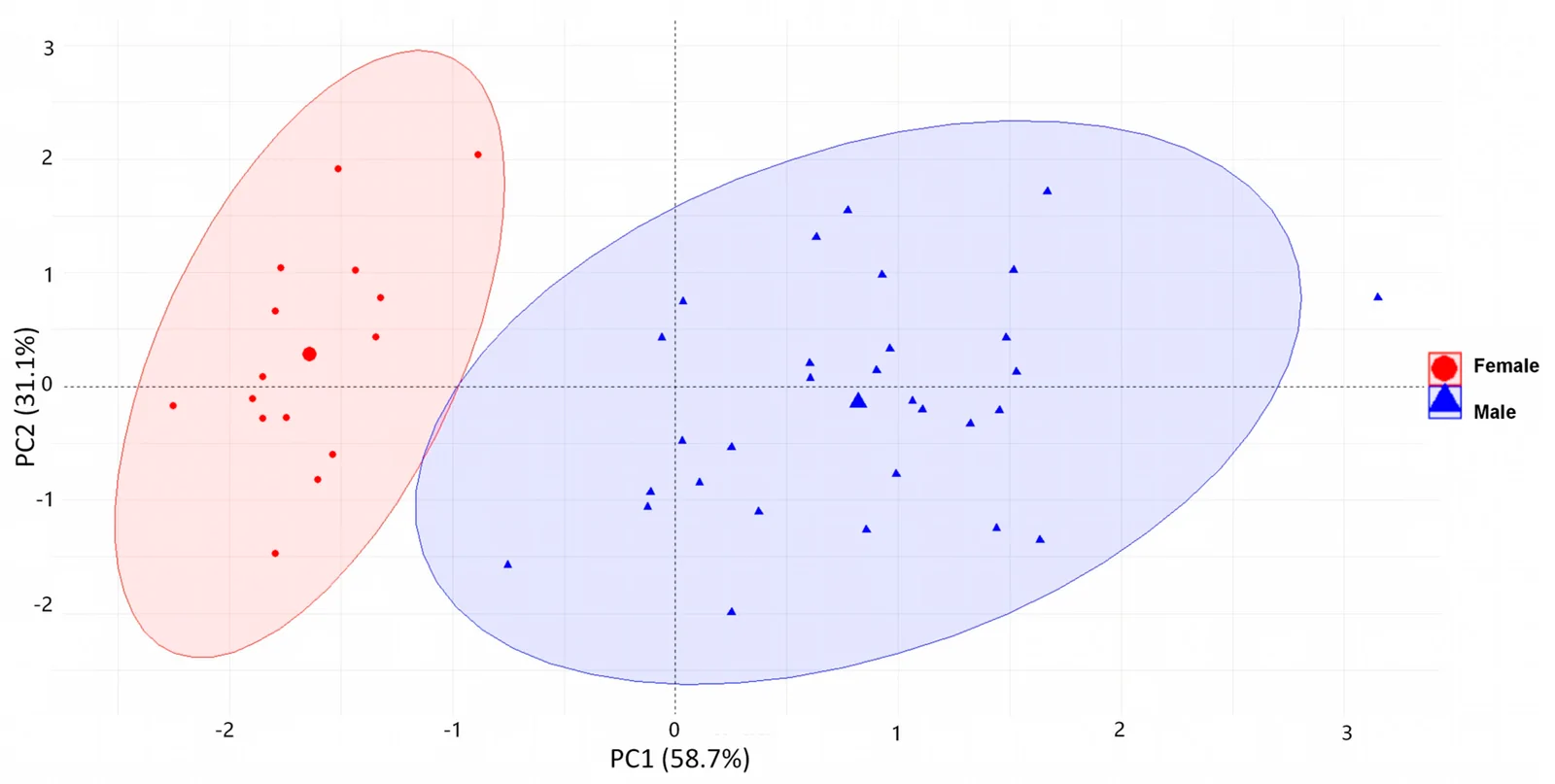
Principal component analysis of floral morphological traits in female (red) and male (blue) flowers. Ellipses represent 95% confidence intervals. PC1 explains 58.7% of total variance; PC2 explains 31.1%.

To assess the multivariate morphological differentiation between sexes, principal component analysis (PCA) was performed on these three traits. The PCA clearly separated female and male individuals into two non-overlapping clusters (Figure 3), with PC1 explaining 58.7% and PC2 explaining 31.1% of total variance. The strong separation along PC1, which accounted for the majority of the variance, was mainly driven by corolla opening diameter and petal width, the two traits that exhibited the most pronounced sexual differences. Together, these two principal components captured nearly 90% of the total morphological variation, indicating that sexual dimorphism in floral traits is primarily reflected in corolla size and petal dimensions rather than in petal length.

### 3.3. Genome-Wide Identification and Characterization of the SgTPS Gene Family

**Figure 4:**
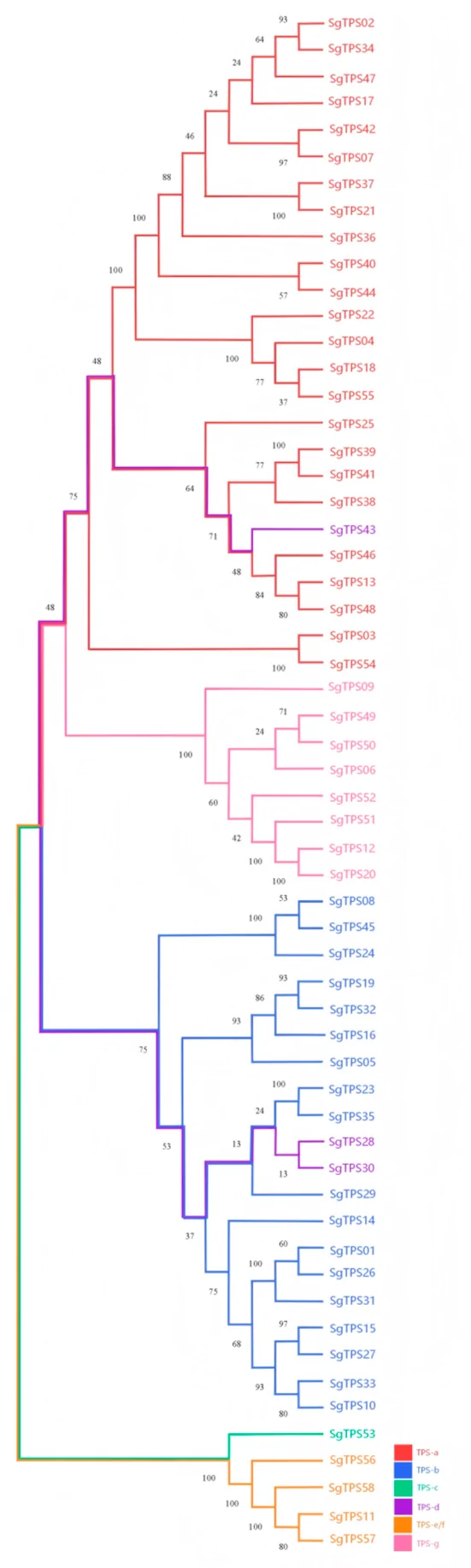
Phylogenetic classification of SgTPS genes. The maximum likelihood tree was constructed with 1000 bootstrap replicates. Six subfamilies are indicated by different colors.

A total of 58 SgTPS genes were identified in the *S. grosvenorii* genome (Supplementary Table S1), a number higher than those reported in *Arabidopsis* (32), tomato (44), and cucumber (28), suggesting lineage-specific expansion [5,13]. Subcellular localization prediction indicated that the vast majority of SgTPS proteins are targeted to the cytoplasm or chloroplasts. All SgTPS proteins were predicted to be hydrophilic (GRAVY < 0), and 89.7% were acidic (pI < 7), with an average pI of 5.68. Phylogenetic analysis classified the 58 SgTPS proteins into six subfamilies: TPS-a (24 members), TPS-b (18), TPS-c (1), TPS-d (3), TPS-e/f (4), and TPS-g (8) (Figure 4). The TPS-a and TPS-b subfamilies together accounted for 72.4% of all members.

**Figure 5:**
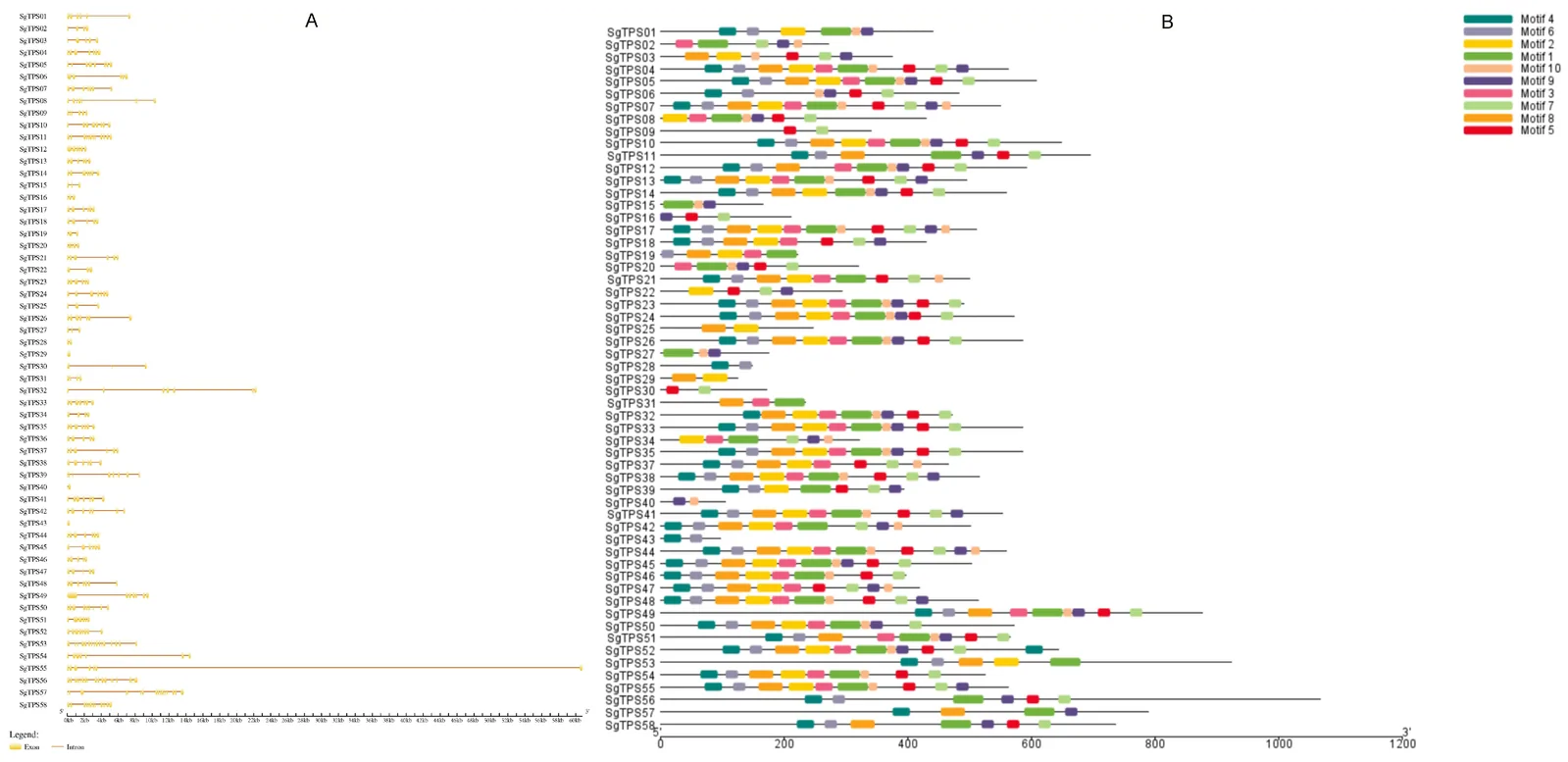
Gene structures and conserved motifs of SgTPS proteins. (**A**) Exon–intron organization of the 58 SgTPS genes. Exons are represented by boxes, introns by lines. (**B**) Distribution of 10 conserved motifs identified by MEME analysis. Detailed motif sequences are provided in Supplementary Table S2.

Gene structure analysis showed that members of the same subfamily shared similar exon–intron patterns, with exon numbers ranging from 3 to 14 across the family (Figure 5A). Notably, TPS-a and TPS-b members typically contained 6–8 exons, whereas TPS-g members had a more compact structure with 5–7 exons. This structural conservation within subfamilies is consistent with their phylogenetic clustering and supports the functional divergence among different TPS clades. Conserved motif analysis identified 10 distinct motifs across SgTPS proteins (Figure 5B; Supplementary Table S2). All SgTPS proteins contained the canonical DDXXD motif (Motif 1) and the DXDD motif (Motif 3), which are necessary for divalent metal ion binding and catalytic activity. Additionally, the RRX_8_W motif (Motif 2), characteristic of monoterpene synthases, was identified in all TPS-b and TPS-g members, additionally supporting their functional annotation. The NSE/DTE motif (Motif 4), involved in cyclization reactions, was conserved across all subfamilies except TPS-c, denoting its key role in terpene skeletal diversification.

**Figure 6:**
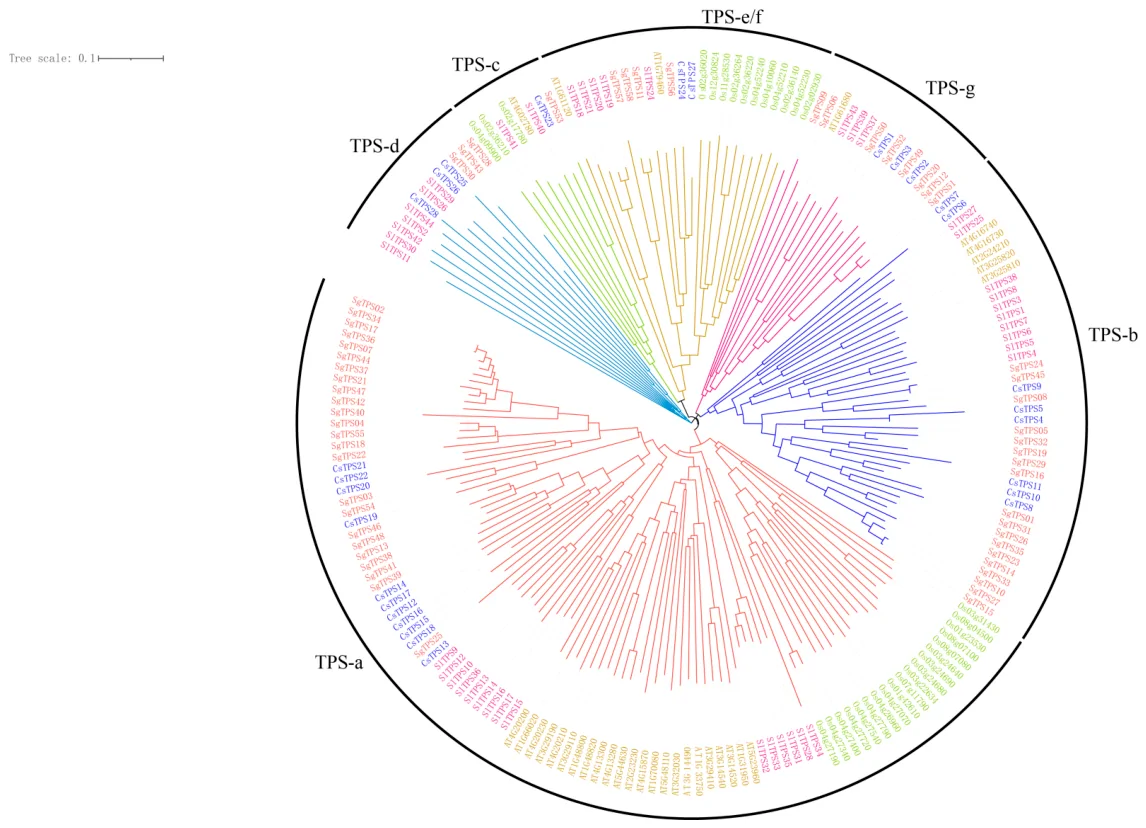
Multi-species phylogenetic analysis of TPS proteins from *S. grosvenorii*, *Cucumis sativus*, *Arabidopsis thaliana*, *S. lycopersicum*, and *Oryza sativa*. The neighbor-joining tree was constructed using the Poisson model with 1000 bootstrap replicates. Different colored arcs represent different species. The six TPS subfamilies (TPS-a, TPS-b, TPS-c, TPS-d, TPS-e/f, and TPS-g) are indicated by background shading. Bootstrap values > 70% are shown at major nodes.

To further elucidate the evolutionary relationships among SgTPS genes within a broader phylogenetic context, a multi-species phylogenetic tree was constructed using TPS protein sequences from *S. grosvenorii*, cucumber, *Arabidopsis*, tomato, and rice (Figure 6). The resulting circular phylogram revealed that SgTPS genes clustered most closely with those of cucumber, consistent with their close taxonomic relationship within the Cucurbitaceae. Notably, the TPS-a and TPS-b subfamilies, which accounted for the majority of SgTPS members, formed distinct clades that were well supported by bootstrap values (>70%), indicating their independent evolutionary histories. In contrast, TPS-c, TPS-e/f, and TPS-g members were interspersed among species, suggesting that these subfamilies may have originated before the divergence of the major angiosperm lineages.

Promoter cis-element analysis showed that SgTPS genes are enriched in elements responsive to phytohormones, light, and abiotic stresses. A comprehensive heatmap of all identified cis-regulatory elements across the 58 SgTPS genes is provided in Supplementary Figure S2. Among the hormone-responsive elements, ABRE (abscisic acid responsiveness) and MeJA (methyl jasmonate responsiveness) motifs were the most abundant, consistent with the involvement of terpenoid metabolism in fruit development and defense responses. The relationship between hormone-responsive cis-elements and expression patterns is examined in Section 3.4 and Section 3.9.

### 3.4. Tissue-Specific Expression Patterns of SgTPS Genes

RNA-seq analysis across leaves, 3 DPP fruits, and 20 DPP fruits showed clear expression clusters among the 33 SgTPS genes with detectable expression. Most SgTPS genes showed elevated relative expression at 3 DPP, with eight genes (*SgTPS03*, *SgTPS04*, *SgTPS07*, *SgTPS09*, *SgTPS11*, *SgTPS14*, *SgTPS31*, and *SgTPS33*) meeting the predefined |log_2_FC| > 1 criterion for classification as 3-DPP-preferential (Cluster I). This early-fruit expression pattern suggests that these genes may be involved in the initial stages of fruit set and early terpenoid metabolism immediately following pollination.

**Figure 7:**
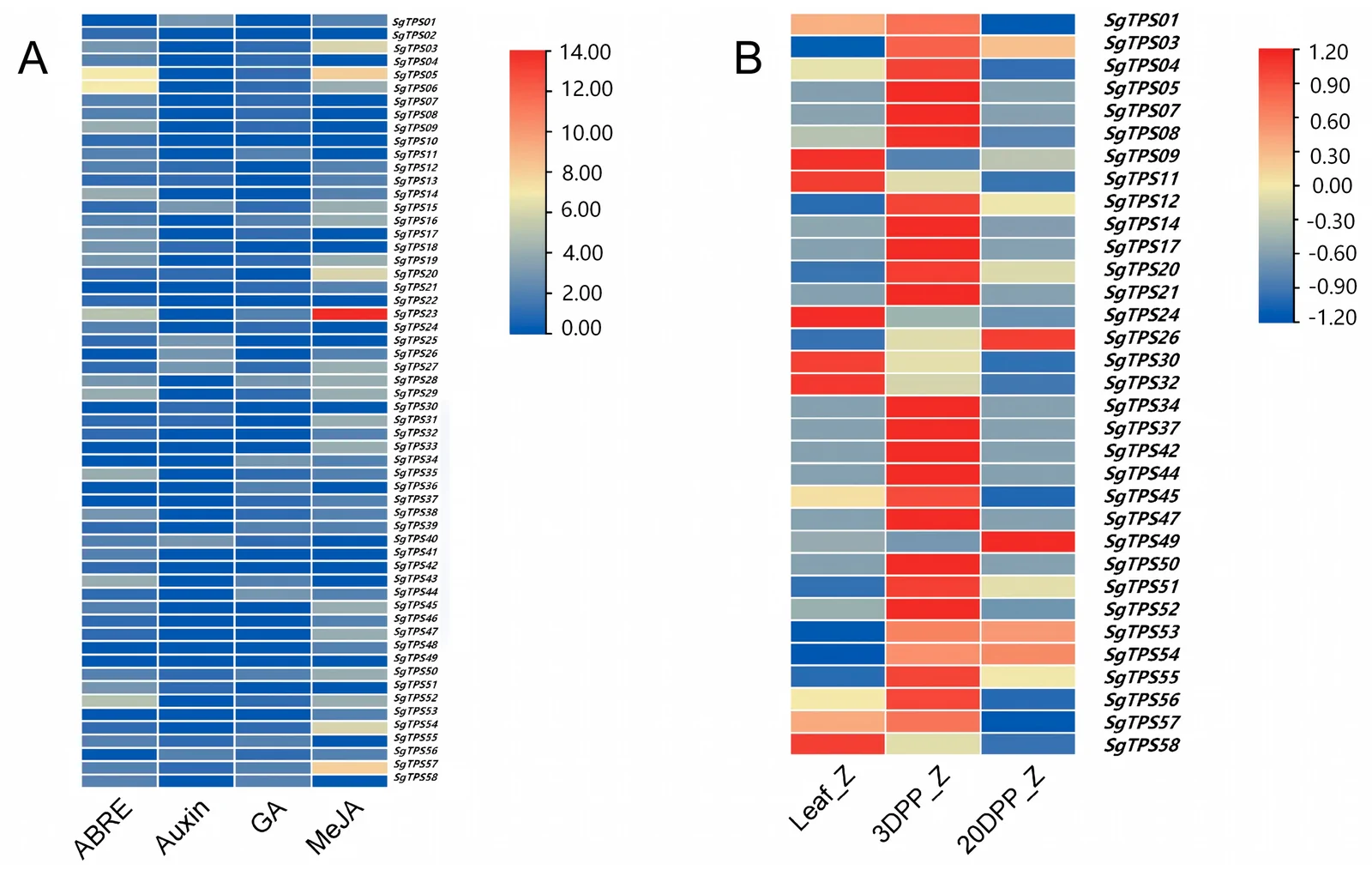
Integrated analysis of cis-regulatory elements and expression patterns of SgTPS genes. (**A**) Distribution of key hormone-responsive cis-elements (ABRE, MeJA, Auxin, GA) in the 2000 bp promoter regions of the 58 SgTPS genes. Rows are clustered by element abundance. (**B**) Z-score normalized expression profiles of the 33 detectably expressed SgTPS genes across leaves, 3 DPP fruits, and 20 DPP fruits. Rows are clustered by expression pattern. Clusters I and II refer to distinct expression patterns detailed in the Results. Raw FPKM values are provided in Supplementary Table S7.

In contrast, *SgTPS49* displayed a strong expression bias toward 20 DPP (Cluster II), with a mean FPKM of 5345.5 at this stage, compared to 2443–4063 in 3-DPP fruits and 2785–4072 in leaves. Although absolute FPKM values were moderate across tissues, Z-score normalization revealed a clear upregulation of *SgTPS49* during rapid fruit expansion relative to earlier stages. This temporal specificity distinguishes *SgTPS49* from the 3-DPP-preferential genes and suggests its involvement in late-stage terpenoid metabolism, rather than in early fruit set. To integrate regulatory and expression data, the distribution of key hormone-responsive cis-elements in the promoters of all 58 SgTPS genes was visualized alongside expression profiles for the 33 genes with detectable expression (Figure 7). *SgTPS26* also exhibited a Z-score profile with slightly higher expression at 20 DPP, though its absolute FPKM values showed only minor variation across tissues (877–962), underscoring that Z-score normalization may exaggerate small absolute changes.

The distinct temporal partitioning of TPS expression, with one set of genes peaking at 3 DPP and *SgTPS49* showing a 20-DPP-biased pattern, suggests a coordinated developmental program in which different terpene synthase members are deployed at successive phases of fruit development. Moreover, comparison of the 3 DPP and 20 DPP transcriptomes identified 12 additional TPS genes whose expression changed by more than two-fold between these stages (Supplementary Table S7), suggesting that terpene metabolism is broadly reprogrammed during fruit development.

### 3.5. Tandem Duplication Events

Manual inspection of the chromosomal locations of the 58 SgTPS genes revealed extensive tandem duplication events. A total of 38 genes were organized into 14 tandem arrays distributed across 10 contigs (Supplementary Table S4). The largest clusters included a four-gene cluster on tig00007658, tig00153348, and tig00153874. Among tandemly duplicated genes, the TPS-a subfamily contributed the most, with 18 genes accounting for 47.4%, followed by TPS-b and TPS-g. Notably, all TPS-d members were involved in tandem duplications, whereas TPS-c was the only subfamily without a tandem duplication event.

**Figure 8:**
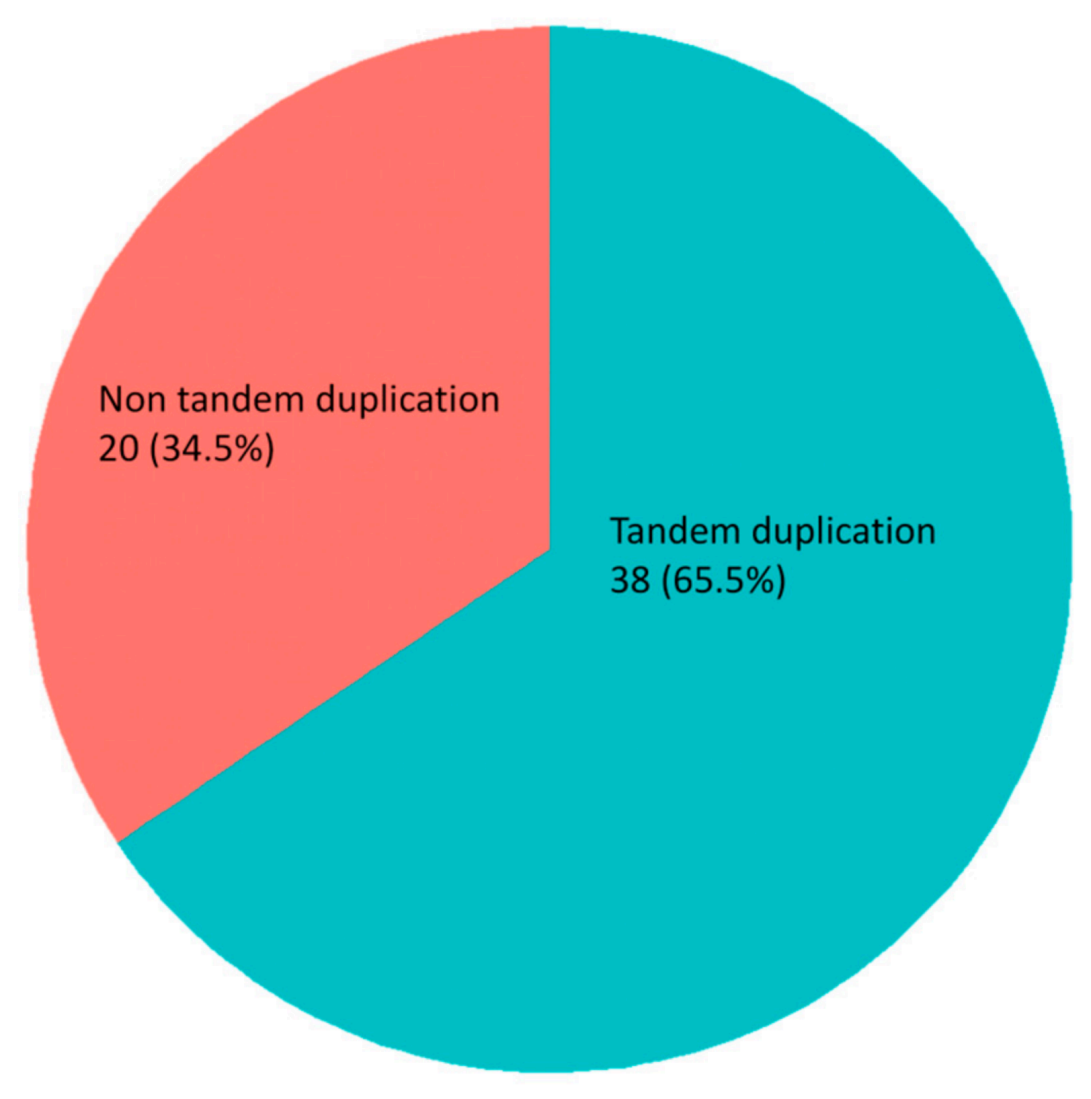
Distribution of duplication types among the 58 SgTPS genes. Red, tandem duplication; blue, other duplication types.

The physical proximity of tandemly duplicated genes, particularly the four-gene clusters, suggests that these loci may represent hotspots of recent duplication activity [37]. The predominance of TPS-a members in tandem arrays is consistent with the overall expansion of this subfamily in the *S. grosvenorii* genome, while the complete absence of tandem duplications in TPS-c suggests that this subfamily may be maintained as a single-copy gene under purifying selection. These results show that tandem duplication has played a dominant role in the expansion and diversification of the SgTPS gene family, particularly in the TPS-a, TPS-g, and TPS-d subfamilies, consistent with observations in other plants where tandem duplications have been shown to drive TPS family expansion. As illustrated in Figure 8, tandem duplication events were identified in 38 of the 58 SgTPS genes (65.5%), while the remaining 20 genes (34.5%) lacked tandem duplication and may have originated from other mechanisms, such as whole-genome duplication or transposed duplication.

### 3.6. Predicted Protein–Protein Interaction Network for SgTPS49

**Figure 9:**
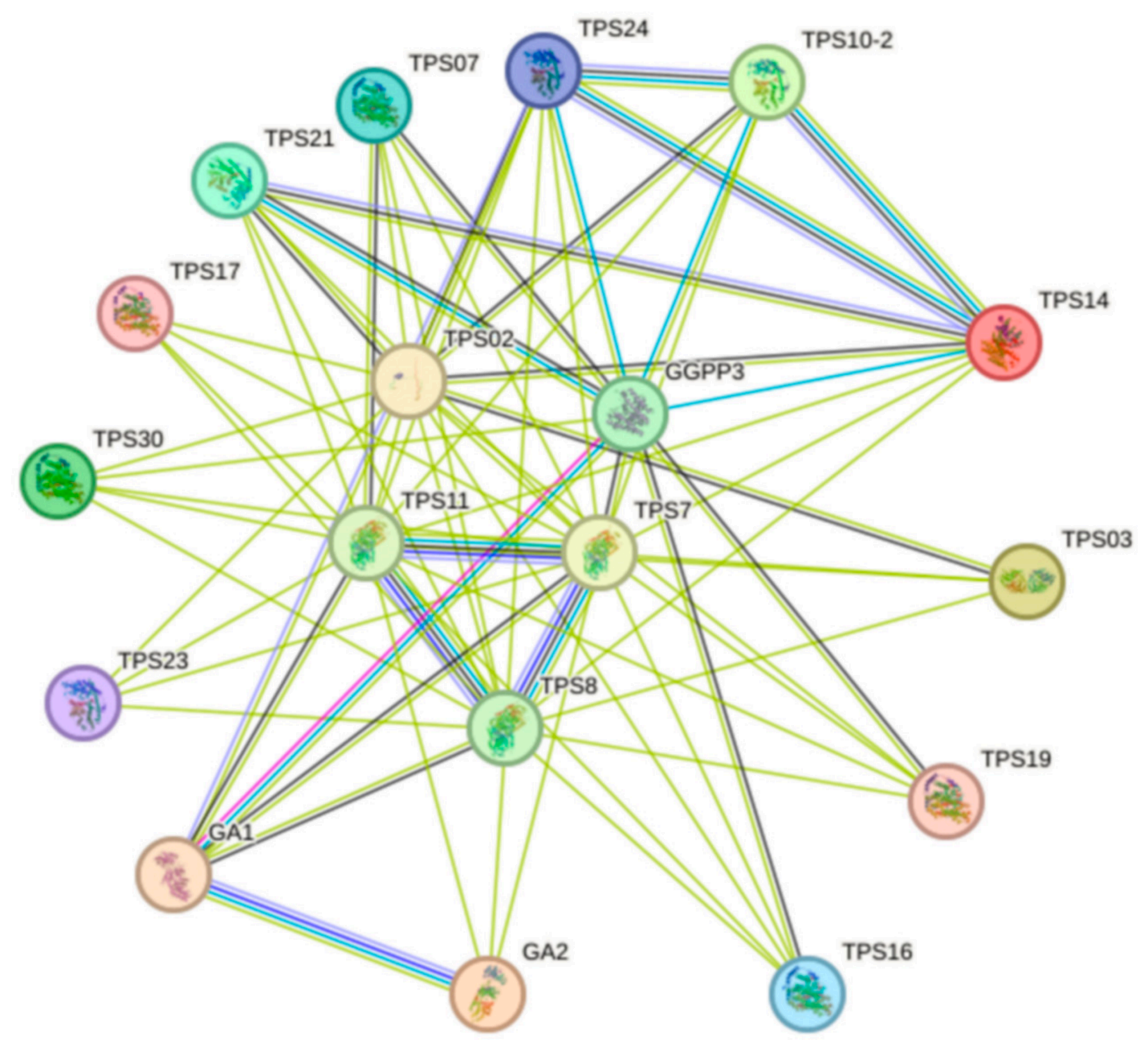
Predicted protein–protein interaction network of SgTPS49 based on STRING analysis. Nodes represent proteins; edges represent predicted functional associations (confidence ≥ 0.70), with thicker lines indicating higher confidence.

A protein–protein interaction network was constructed using STRING based on the top *Arabidopsis* homolog AT1G23870.1. The predicted network suggested that SgTPS49 may interact directly or indirectly with several key enzymes in the upstream methylerythritol phosphate (MEP) and mevalonate (MVA) pathways, including 1-deoxy-D-xylulose-5-phosphate synthase (DXS) and 3-hydroxy-3-methylglutaryl coenzyme A synthase (HMGS). Furthermore, potential interactions were predicted with transcription factors, including MYB and bHLH proteins (Figure 9). Full STRING homology mapping for all 58 SgTPS genes is provided in Supplementary Table S6. The potential involvement of bHLH transcription factors in regulating terpene biosynthesis has been reported in several plant species.

### 3.7. In Silico Structural and Docking Analysis of SgTPS49

**Figure 10:**
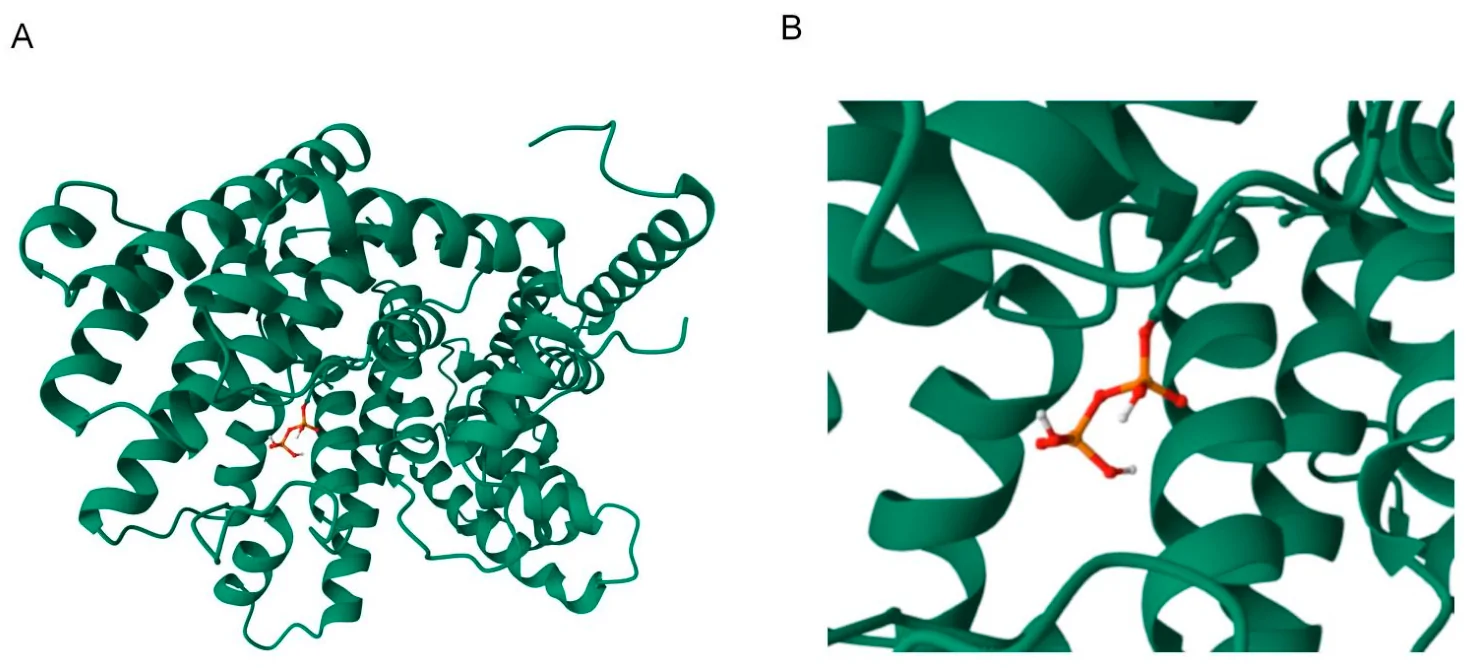
Homology modeling and molecular docking of SgTPS49 with geranyl diphosphate (GPP). (**A**) Three-dimensional structure of SgTPS49 showing the conserved DDXXD motif in the active site cavity. (**B**) Binding pose of GPP within the predicted active site.

The three-dimensional structure of SgTPS49 was predicted using SWISS-MODEL [38], with the AlphaFold DB model of *Cucurbita moschata* nerolidol synthase 1-like serving as the template. The resulting model showed a canonical terpene synthase α-helical fold, with the conserved DDXXD motif positioned within a solvent-accessible cavity (Figure 10A). Cavity-guided blind docking using CB-Dock2 [39] identified a high-confidence binding cavity with a favorable Vina score of −7.1 kcal/mol for geranyl diphosphate (GPP), notably more stable than the five additional cavities identified by structure-based blind docking, which yielded scores ranging from −5.9 to −6.4 kcal/mol (Supplementary Table S5-1). The docking pose revealed that the diphosphate moiety of GPP is coordinated by the aspartate residues of the DDXXD motif (Asp631 and Asp635) via predicted Mg^2+^-mediated interactions, while the hydrocarbon tail extends into a hydrophobic pocket lined by residues including Ile624, Val734, and Leu771 (Figure 10B). The contact residues within 4 Å of GPP are listed in Supplementary Table S5-2. Key residues forming hydrogen bonds and hydrophobic contacts with GPP are detailed in Supplementary Table S5-3.

This binding configuration is consistent with that of characterized members of the TPS-g subfamily [7,10], suggesting that SgTPS49 may function as a monoterpene synthase.

### 3.8. Synteny Analysis of SgTPS Genes

**Figure 11:**
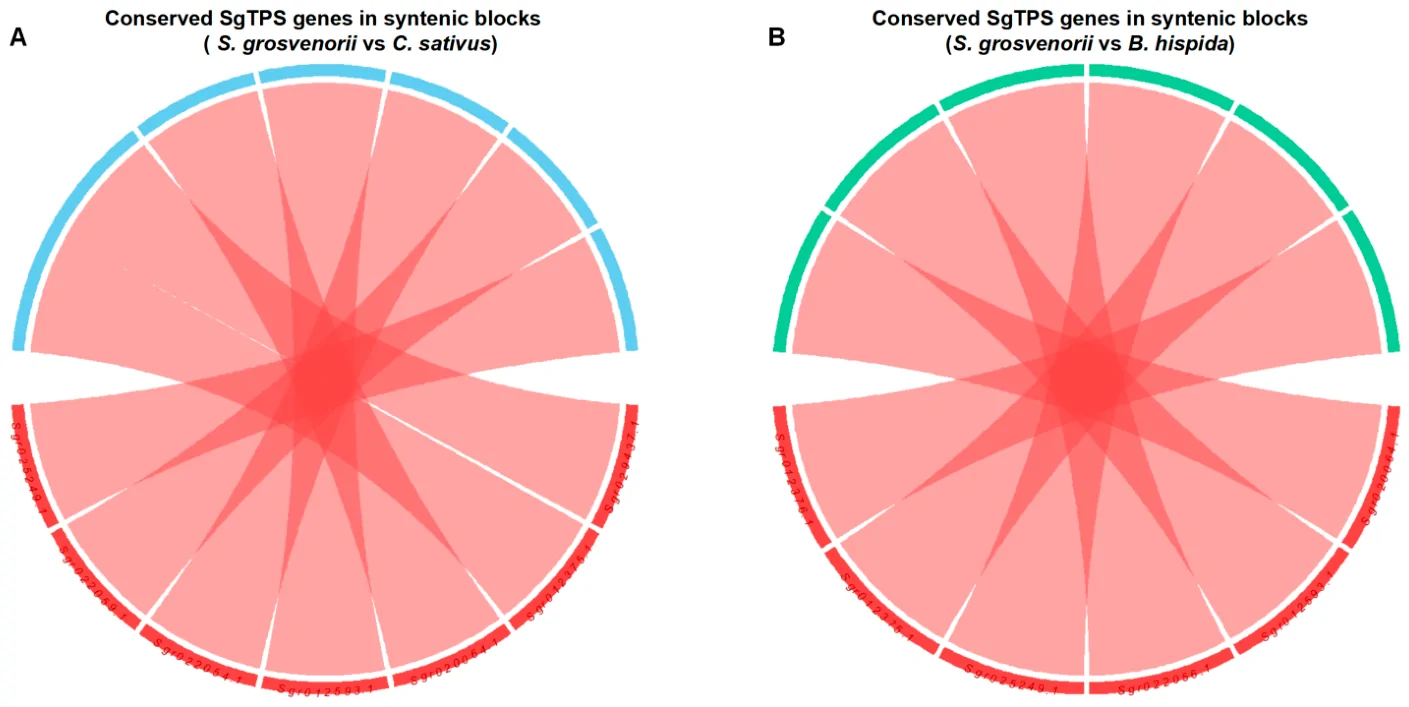
Synteny conservation of SgTPS genes between *S. grosvenorii* and representative cucurbit species. Chord diagrams showing conserved SgTPS genes and their syntenic counterparts in (**A**) cucumber and (**B**) wax gourd. Red sectors represent syntenic SgTPS genes; blue/green sectors represent their respective orthologs. Connecting lines indicate syntenic gene pairs.

To investigate the evolutionary conservation of the SgTPS gene family within the Cucurbitaceae, comparative synteny analyses were performed between *S. grosvenorii* and two representative cucurbit species, cucumber (*C. sativus*) and wax gourd (*B. hispida*). Genome-wide synteny blocks were identified using MCScanX [41] with an E-value threshold of 1 × 10^−5^. Extensive genome-wide collinearity was observed between *S. grosvenorii* and both cucurbit species, confirming a shared ancestral karyotype (Figure 11).

However, only 7 of the 58 SgTPS genes (*SgTPS09*, *SgTPS25*, *SgTPS38*, *SgTPS43*, *SgTPS49*, *SgTPS54*, and *SgTPS56*) were anchored within syntenic blocks shared with cucumber. Similarly, only six genes (*SgTPS09*, *SgTPS12*, *SgTPS25*, *SgTPS39*, *SgTPS54*, and *SgTPS56*) were located in conserved blocks with wax gourd (Figure 11). A core set of five genes (*SgTPS09*, *SgTPS25*, *SgTPS54*, and *SgTPS56*, plus one species-specific member in each comparison) was conserved across both comparisons, likely representing the ancestral TPS repertoire in the Cucurbitaceae. The remaining 51–52 SgTPS genes, representing approximately 88% of the family, showed no syntenic conservation with either cucumber or wax gourd. These non-syntenic genes were predominantly from the TPS-a and TPS-b subfamilies, the most significantly expanded clades in the SgTPS repertoire.

### 3.9. Association Between Promoter Cis-Elements and Expression Patterns

To explore the possible regulatory basis underlying the distinct expression patterns of SgTPS genes, the distribution of major hormone-responsive cis-elements (ABRE, MeJA, Auxin, and GA) in the 2000 bp promoter regions was integrated with the transcriptome data. The integrated visualization of hormone-responsive cis-elements and expression patterns is presented in Figure 7 (see Section 3.4). Among the eight 3-DPP-preferential genes, several contained more MeJA- and ABRE-responsive elements than the genome-wide average (mean 2.3 MeJA elements per promoter). For instance, *SgTPS03*, *SgTPS31*, and *SgTPS33* each harbored 4–6 MeJA-responsive motifs, while *SgTPS09* and *SgTPS14* each contained 3–4 ABRE motifs. Jasmonate and abscisic acid signaling are known regulators of early fruit set, abscission, and wound response, consistent with the peak expression of these genes shortly after pollination. However, the element enrichment was not uniform across all eight genes. Notably, *SgTPS04* and *SgTPS07* contained relatively few hormone-responsive elements, suggesting that additional transcriptional regulators may contribute to their coordinated expression at 3 DPP. Notably, *SgTPS23*, a gene not classified as 3-DPP-preferential, harbored 14 MeJA-responsive elements, the highest count among all 58 SgTPS genes, denoting strong potential for jasmonate-mediated regulation.

Strikingly, *SgTPS49*, which was specifically upregulated at 20 DPP, possessed a promoter virtually devoid of the four major hormone-responsive elements analyzed (Figure 7A). This complete absence suggests that the 20-DPP-specific expression of *SgTPS49* is unlikely to be directly mediated by ABA, MeJA, auxin, or GA signaling. Instead, its regulation may involve developmental transcription factors (e.g., MYB, bHLH, or NAC family members), epigenetic modifications, or distal regulatory elements that are not captured within the 2000 bp proximal promoter. This unique regulatory architecture distinguishes *SgTPS49* from the 3-DPP-preferential SgTPS genes and highlights it as an interesting candidate for promoter-focused functional dissection.

The remaining SgTPS genes, most of which were not detectably expressed in the three tissues analyzed, showed no obvious correlation between hormone-responsive element abundance and expression status. Their transcriptional silencing may involve repressive chromatin states or the absence of necessary activating signals. These observations provide testable hypotheses for future studies to investigate the transcriptional regulation of *SgTPS49* and other SgTPS genes.

## 4. Discussion

This study presents the first comprehensive analysis of sexual dimorphism and the *TPS* gene family in *S. grosvenorii*, integrating floral phenotypic divergence, genome-wide identification and evolutionary dynamics of the SgTPS family, and in silico characterization of a candidate fruit-specific enzyme.

### 4.1. Floral Sexual Dimorphism and Pollinator Attraction

The coordinated dimorphism in floral morphology and volatile emission strongly suggests differential pollinator attraction as a reproductive strategy in *S. grosvenorii*, consistent with patterns noted in other dioecious species [28,29]. The male-specific sesquiterpene burst during mid-morning coincides with the peak foraging activity of many insect pollinators, as reported in other dioecious species. Our observation of significantly greater variation in male corolla opening and volatile composition, contrasted with the highly conserved monoterpene signals and morphology of female flowers, aligns with the theory of sexual selection in plants: male fitness depends on multiple pollinator visits for sufficient pollen export, selecting for showier and more variable floral displays, whereas female fitness can be achieved through one or a few effective visits, favoring consistent signals that ensure efficient pollen transfer [28,29]. The conservation of petal length between sexes further supports the idea that both sexes share a common pollinator attractant function for this trait, whereas corolla size and scent composition have diverged to optimize sex-specific reproductive tactics. Although alternative explanations, such as sex-specific defense strategies or resource assignment trade-offs, cannot be ruled out, the statistically significant differences in both morphological traits and volatile composition support a pollinator-mediated selection scenario.

The predominance of α-pinene and β-pinene in female flowers is noteworthy given the well-documented ecological functions of these monoterpenes. α-Pinene and β-pinene serve as potent olfactory signals for a broad range of insect pollinators, including bees and moths, and have been shown to act as airborne defense compounds against floral herbivores and pathogens [42,43]. In *S. grosvenorii*, the sustained emission of these pinenes by female flowers throughout the day may serve a dual ecological function: (i) providing a reliable, long-range attractant for pollinators, thereby reducing the risk of pollination failure, and (ii) conferring constitutive chemical defense to protect the pistil and developing ovules during the extended receptive period. The highly conserved pinene signals in female flowers, contrasted with the more variable and complex sesquiterpene-rich profiles in male flowers, suggest sex-specific optimization of these ecological functions under pollinator-mediated selection. This is consistent with the broader view of floral volatiles as multifunctional signals that mediate both mutualistic and antagonistic interactions, as reviewed by Dötterl and Gershenzon [42] and Pichersky and Gershenzon [43].

### 4.2. SgTPS Gene Family Expansion and Evolutionary Dynamics

The multi-species phylogenetic analysis placed SgTPS genes within a wider evolutionary framework. The close clustering of SgTPS genes with cucumber TPS genes (Figure 6) confirmed their phylogenetic affinity within the Cucurbitaceae and provided a phylogenetic basis for the subsequent synteny comparisons. This clustering pattern is consistent with the TPS family organization reported in cucumber [19], where TPS-a and TPS-b subfamilies also dominate the repertoire, suggesting a shared ancestral TPS expansion in the Cucurbitaceae lineage. The distinct division of TPS-a and TPS-b clades, with high bootstrap support, further suggests that these subfamilies have undergone independent and extensive diversification in *S. grosvenorii*, probably driven by the tandem duplication events described below.

Notably, the sex-specific volatile profiles established in flowers (Figure 1) are temporally correlated with distinct patterns of TPS gene activation during subsequent fruit development (Figure 7). While female flowers consistently emit monoterpenes throughout the day, the 3-DPP-preferential activation of multiple TPS genes (Cluster I, Figure 7B) suggests that early fruit set immediately following pollination represents a critical phase for terpenoid metabolism. This temporal partitioning highlights a coordinated deployment of TPS family members across reproductive stages, where floral volatiles mediate pollinator attraction and early-fruit TPS expression potentially contributes to fruit set or defense. Similar functional coordination between floral and fruit terpenoid metabolism has been documented in tomato, where distinct TPS clades contribute to flower fragrance and fruit flavor, respectively [5], and in kiwifruit, where TPS genes exhibit overlapping functions in fruit aroma and floral bouquet [44].

The expansion of the SgTPS family to 58 members, chiefly within the TPS-a and TPS-b subfamilies, which together account for 72.4% of all members, indicates the complexity of terpenoid metabolism in this species. This family size is notably larger than that of many other sequenced plants, such as *Arabidopsis* (32) or cucumber (28), and comparable to that of tomato (44), a well-known model for fruit volatile studies. These findings highlight strong evolutionary pressure for the diversification of terpenoid profiles in monk fruit. Of particular relevance to our findings, the TPS-a subfamily, which is primarily responsible for sesquiterpene biosynthesis, has undergone substantial expansion in several plant species, as demonstrated by the genome-wide characterization of TPS-a members in *Medicago truncatula* [45], suggesting that TPS-a expansion may be a common evolutionary strategy for diversifying specialized metabolism.

This expansion pattern mirrors observations in other plants, where tandem duplications have rapidly driven lineage-specific expansion of TPS genes across angiosperms [37,46]. Notably, the fact that all TPS-d members were tandemly duplicated points to a recent burst of this subfamily, whereas the solitary TPS-c member, *SgTPS53*, appears to follow a separate evolutionary course, suggesting differential adaptive pressures acting across subfamilies.

This lineage-specific expansion model is powerfully supported by our synteny analyses with cucumber and wax gourd. While extensive genome-wide collinearity confirms their common ancestry within the Cucurbitaceae, the finding that ~88% of SgTPS genes fall outside any conserved syntenic block is striking. This indicates that the majority of the SgTPS repertoire originated after the divergence of *Siraitia* from these other cucurbit lineages. The combined duplication and synteny data support an evolutionary model in which an ancestral core of five TPS genes (*SgTPS09*, *SgTPS25*, *SgTPS54*, and *SgTPS56*, plus one species-specific member in each comparison) has been retained in conserved genomic positions since the divergence of the Cucurbitaceae, while the TPS-a and TPS-b subfamilies have undergone explosive, lineage-specific expansion predominantly through tandem duplication events. This independently evolved TPS toolkit likely provided the genetic substrate for the biosynthesis of mogrosides, sex-specific floral volatiles, and other specialized terpenoids unique to *S. grosvenorii*. Analogous lineage-specific TPS expansions driven by tandem duplication have been reported in other medicinal plants, such as the chromosome-level genome analysis of *Atractylodes lancea* [47], further supporting the role of tandem duplication in diversifying terpenoid metabolism in medicinal species.

The high number of TPS-a genes (24) is especially notable, as they are primary consumers of FPP, the same precursor required for mogroside biosynthesis. We speculate that this expansion could create a metabolic sink competing with mogroside accumulation, possibly contributing to variations in mogroside accumulation [24]; empirical confirmation through FPP metabolic flux analysis is warranted. Furthermore, enzymes upstream of mogroside biosynthesis, such as squalene epoxidases, have been functionally characterized in *S. grosvenorii* [48], highlighting the complexity of its terpenoid metabolic network. Previous work on cucurbit terpenoid metabolism, such as the characterization of sesquiterpene biosynthesis in melon rinds [20], provides a useful comparative framework for understanding the metabolic context of TPS-mediated terpenoid diversity in monk fruit.

### 4.3. Regulatory Divergence and the Unique Case of SgTPS49

The integrated promoter–expression analysis shows an unexpected dichotomy in regulatory strategies within the SgTPS family. The 3-DPP-preferential genes appear to be largely regulated by classical hormone pathways, particularly jasmonate and abscisic acid, known regulators of early fruit development, abscission, and defense [4,5]. The extreme case of *SgTPS23*, with its 14 MeJA motifs, represents a potential hotspot for jasmonate-mediated regulation, although its expression pattern suggests this potential may be context-dependent or demand a specific trigger.

**Figure 12:**
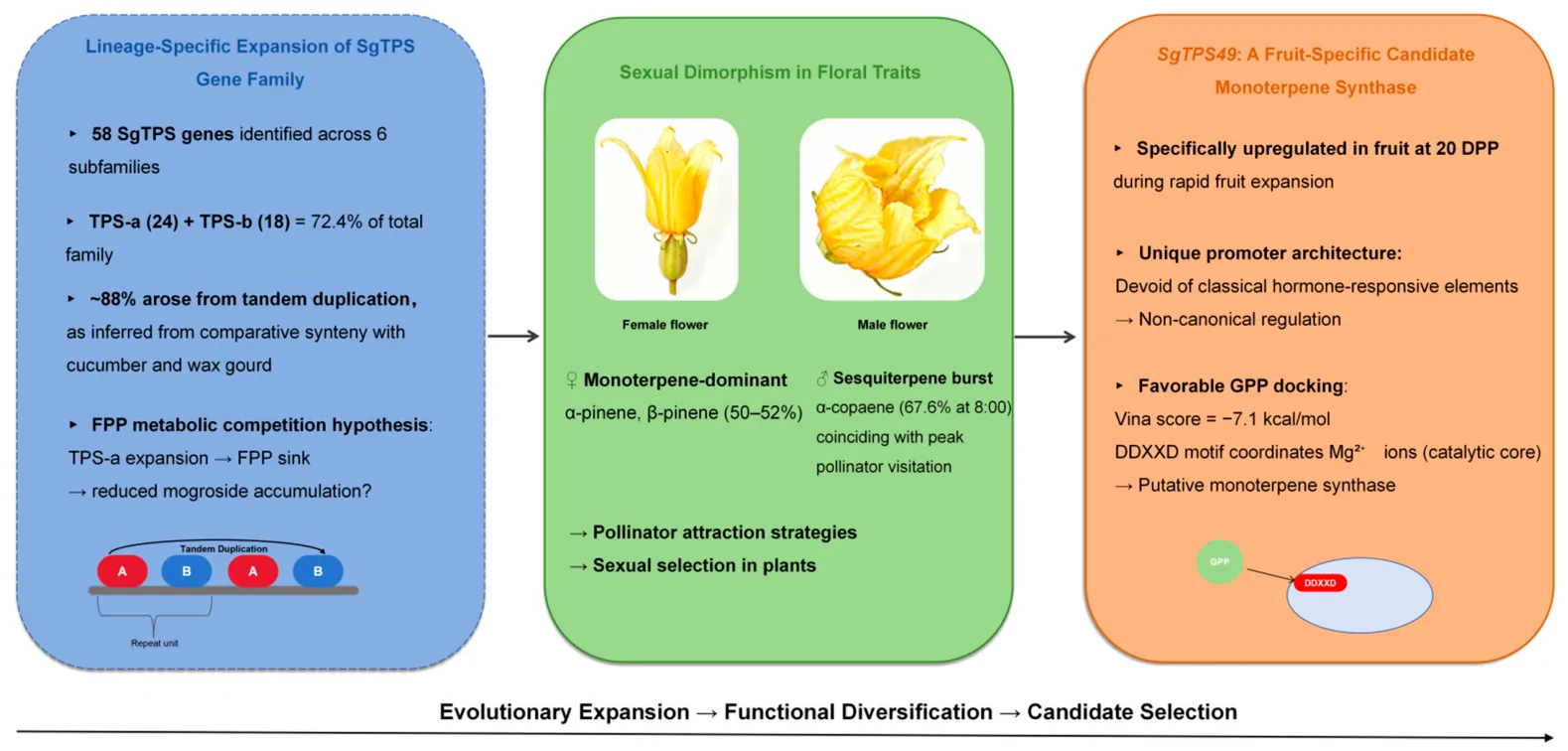
Hypothetical working model of the SgTPS family expansion, sexual dimorphism, and *SgTPS49*-mediated terpenoid metabolism in *S. grosvenorii*. (**Left**) Lineage-specific expansion of the SgTPS family: 58 members across six subfamilies, with TPS-a (24) and TPS-b (18) comprising 72.4% of the total, and approximately 88% arising from tandem duplication, as inferred from comparative synteny with cucumber and wax gourd. The red rounded rectangles with “A” and blue rounded rectangles with “B” represent Gene A and Gene B, respectively, and their alternating arrangement illustrates the multi-copy gene array structure formed by tandem duplication, potentially creating an FPP metabolic sink that may indirectly affect mogroside biosynthesis. (**Middle**) Sexual dimorphism in floral traits: female flowers exhibit smaller corolla openings and monoterpene-dominant scents, whereas male flowers display larger corollas with greater morphological variation and a mid-morning sesquiterpene burst, consistent with differential pollinator attraction strategies in dioecious species. (**Right**) *SgTPS49* as a fruit-specific candidate: specifically upregulated in fruit at 20 DPP during rapid fruit expansion, with a unique promoter architecture devoid of classical hormone-responsive elements, and favorable GPP docking (Vina score = −7.1 kcal/mol) supporting its annotation as a putative monoterpene synthase. Arrows indicate the proposed evolutionary and functional trajectory from gene family expansion to candidate gene selection. Abbreviations: DPP, days post-pollination; FPP, farnesyl diphosphate; GPP, geranyl diphosphate; TPS, terpene synthase.

The most compelling finding is the unique regulatory architecture of *SgTPS49*. As the only 20-DPP-biased gene, its specificity to the rapid fruit expansion stage is consistent with its possible role in synthesizing volatile terpenoids that contribute to fruit flavor or act as developmental signals during the period of active mogroside accumulation [18]. However, its promoter is a “blank slate” for the four major hormone pathways analyzed, completely lacking ABRE, MeJA, Auxin, and GA elements. This strongly suggests that its precise temporal regulation is governed by a distinct set of developmental master regulators, such as MYB, bHLH, or NAC transcription factors, whose binding sites may not be captured by our hormone-focused analysis. This hypothesis is consistent with the broader understanding that transcription factors, particularly members of the MYB, bHLH, and NAC families, play central roles in regulating terpenoid biosynthesis in plants, as comprehensively reviewed by Huang et al. [49]. This scenario corresponds to the ripening-specific regulation of TPS genes in tomato by master transcription factors, including RIN and NOR [4,5]. The predicted protein–protein interaction network for SgTPS49, showing connections to both upstream terpenoid pathway enzymes and MYB/bHLH factors, provides further support for this hypothesis. This integrative strategy of coupling transcriptomic profiling with protein–protein interaction assays has recently been employed to resolve the regulatory roles of NAC transcription factors in *Taraxacum kok-saghyz*, a non-model rubber-producing plant [50]. Regarding the predicted linalool synthase activity of *SgTPS49* (Supplementary Table S6), we note that linalool was not detected in any of the headspace volatile samples collected from intact female or male flowers across all seven time points (Supplementary Table S3; see also Section 3.1). It is important to clarify that the transcriptome dataset used in this study (Xia et al., 2018 [23]) did not include floral tissues, and thus the expression level of *SgTPS49* in flowers cannot be directly assessed. However, *SgTPS49* was found to be preferentially upregulated at 20 DPP relative to 3 DPP fruits and leaves (Figure 7), suggesting a potential association of its transcriptional regulation with later stages of fruit development. Whether *SgTPS49* is also expressed in floral tissues remains to be determined. The observed absence of linalool in the floral headspace does not preclude its presence in planta, because the HS-SPME method employed in this study captures only freely emitted volatiles from intact flowers, whereas many monoterpene alcohols, including linalool, can be stored as non-volatile glycosylated conjugates or sequestered within specialized structures and released only upon tissue disruption [42]. Furthermore, TPS enzymes often exhibit product promiscuity, generating different terpene skeletons depending on substrate availability (GPP vs. FPP), metal ion cofactors, and cellular context, as reviewed by Bergman and Dudareva [51]. Therefore, the favorable GPP docking (Vina score = −7.1 kcal/mol) supports monoterpene synthase activity, but the specific product profile of *SgTPS49* can only be determined through in vitro enzymatic assays with recombinant protein, which we have identified as a key future direction (Section 4.4). In silico docking supports *SgTPS49* as a putative monoterpene synthase. Together with its specific expression pattern and promoter architecture, these results position *SgTPS49* as a leading candidate for future functional characterization. Uncovering the specific upstream regulators of *SgTPS49* will be a key next step. A hypothetical working model integrating these findings is presented in Figure 12.

### 4.4. Limitations and Future Perspectives

Several limitations of this study should be acknowledged. The RNA-seq dataset from Xia et al. [23] included two biological replicates for fruit tissues (3 DPP and 20 DPP), which may limit the statistical power of standard differential expression testing. However, these data were previously used successfully for DESeq2-based differential expression analysis in the original study [23]. We therefore applied an empirical |log_2_FC| > 1 criterion for candidate gene prioritization to complement the original analysis; future qRT-PCR validation with additional biological replicates is essential to confirm these outcomes. Z-score heatmaps, while useful for visualizing relative expression patterns, can increase small absolute changes (as seen with *SgTPS26*), and must be interpreted alongside raw FPKM values (Supplementary Table S7). Additionally, our promoter analysis was confined to the 2000 bp proximal region, and our synteny analysis included two representative cucurbit species. Future work, including more cucurbit genomes with chromosome-level assemblies, as well as the haplotype-resolved T2T genome for *S. grosvenorii* [24], will enable higher-resolution dissection of SgTPS family expansion. Beyond genomic analyses, the integration of transcriptomic data with targeted physiological and biochemical measurements has provided mechanistic insights into reproductive organ development in perennial crops, as demonstrated in areca palm (*Areca catechu*) [52]. Regarding SgTPS49, the determination of its catalytic product(s) will require in vitro enzyme assays with recombinant protein, heterologous expression in a suitable host system, and functional characterization using targeted metabolomics approaches [6,17,51]. These experiments will be essential to verify whether SgTPS49 functions as a linalool synthase as predicted, or produces alternative terpenoid products depending on substrate availability and cellular context. Functionally, the priority is the in vitro and in vivo validation of *SgTPS49*. Its unique expression pattern and promoter architecture make it a perfect candidate for in vitro enzymatic assays, heterologous expression, and promoter-deletion studies to pinpoint the regulatory elements that control its fruit specificity. Genetic transformation systems and emerging CRISPR/Cas9 technologies offer promising avenues for future functional investigations.

## 5. Conclusions

This study identified 58 TPS genes in the *S. grosvenorii* genome, revealing significant expansion of the TPS-a and TPS-b subfamilies, which formed distinct, well-supported clades in multi-species phylogenetic analyses and expanded primarily through tandem duplication. Pronounced sexual dimorphism was observed in both floral morphology and volatile emissions, supporting pollinator-mediated selection. Expression profiling showed widespread TPS gene activation at 3 DPP, with eight genes classified as 3-DPP-preferential and likely regulated by MeJA and ABA, and a 20-DPP-biased candidate, *SgTPS49*, whose promoter lacks classical hormone-responsive elements. In silico docking supports SgTPS49 as a putative monoterpene synthase with a favorable predicted binding mode for GPP. Comparative synteny analyses with cucumber and wax gourd identified only 7 and 6 SgTPS genes, respectively, within conserved syntenic blocks, indicating that about 88% of the family originated from lineage-specific duplication events. Collectively, these results lay a comprehensive genomic foundation for elucidating terpenoid metabolism in *S. grosvenorii* and identify *SgTPS49* as a promising target for future functional validation.

## Figures and Tables

**Table 1:** Morphological traits of female and male flowers of *S. grosvenorii* (mean ± SD).

Trait	Female (*n* = 15)	Male (*n* = 30)	*p*-Value
Corolla opening diameter (mm)	22.90 ± 1.34	30.82 ± 4.06	<0.0001
Petal length (mm)	35.96 ± 1.14	36.27 ± 1.27	0.43
Petal width (mm)	11.57 ± 0.98	17.60 ± 1.47	<0.0001

## Data Availability

The original genomic and transcriptomic data analyzed in this study are openly available in the European Nucleotide Archive (BioProjects PRJEB23465 and PRJEB23466) and the Genome Sequence Archive (accession CRA000522). The original contributions generated during this study are included in the article and its Supplementary Materials. Further inquiries can be directed to the corresponding author.

## Supplementary Materials

The following supporting information can be downloaded at: https://www.mdpi.com/article/10.3390/genes17080926/s1. Figure S1: Comparison of volatile compound contents in female and male *Siraitia grosvenorii* flowers at 6:00, 8:00, and 10:00; Figure S2: Comprehensive distribution of all cis-regulatory elements (including hormone-responsive, light-responsive, stress-responsive, and development-related elements) in the 2000 bp promoter regions of the 58 SgTPS genes; Table S1: Detailed information of 58 identified SgTPS genes; Table S2: Sequences of the 10 conserved motifs identified in SgTPS proteins; Table S3: Volatile compounds detected in female and male flowers at each time point; Table S4: Tandem duplication events in the SgTPS gene family; Table S5-1: Summary of molecular docking results of SgTPS49 with geranyl diphosphate (GPP); Table S5-2: Contact residues within 4 Å of GPP in the optimal binding cavity of SgTPS49; Table S5-3: Key intermolecular interactions between SgTPS49 and GPP in the optimal binding cavity; Table S6: STRING-based homology mapping and functional annotation of SgTPS genes; Table S7: Differentially expressed SgTPS genes between 3 DPP and 20 DPP fruits (fold change > 2 or stage-specific).

## Abbreviations

The following abbreviations are used in this manuscript:

BLAST	Basic Local Alignment Search Tool
bHLH	Basic Helix–Loop–Helix
CDD	Conserved Domain Database
DPP	Days Post-Pollination
DXS	1-Deoxy-D-xylulose-5-phosphate Synthase
FDR	False Discovery Rate
FPKM	Fragments Per Kilobase per Million mapped reads
GC–MS	Gas Chromatography–Mass Spectrometry
GMQE	Global Model Quality Estimate
GPP	Geranyl Diphosphate
HMMER	Hidden Markov Model-based sequence analysis
HMGS	3-Hydroxy-3-Methylglutaryl Coenzyme A Synthase
HS-SPME	Headspace Solid-Phase Microextraction
MEP	Methylerythritol Phosphate
ML	Maximum Likelihood
MVA	Mevalonate
MYB	Myeloblastosis (transcription factor family)
PCA	Principal Component Analysis
qRT-PCR	Quantitative Real-Time Polymerase Chain Reaction
SD	Standard Deviation
STRING	Search Tool for the Retrieval of Interacting Genes/Proteins
TPS	Terpene Synthase
